# Vaughan-Jackson-like syndrome as an unusual presentation of Kienböck's disease: a case report

**DOI:** 10.1186/1752-1947-5-325

**Published:** 2011-07-25

**Authors:** Tooba Mazhar, Rohit Rambani

**Affiliations:** 1 Department of Orthopaedics, Hull & East Yorkshire Hospitals, NHS Trust, Analaby Road Hull, HU3 2JZ UK

## Abstract

**Introduction:**

Kienböck's disease is a condition of osteonecrosis of the lunate bone in the hand, and most patients present with a painful and sometimes swollen wrist with a limited range of motion in the affected wrist. Vaughan-Jackson syndrome is characterized by the disruption of the digital extensor tendons, beginning on the ulnar side with the extensor digiti minimi and extensor digitorum communis tendon of the small finger. It is most commonly associated with rheumatoid arthritis. We describe a case of a patient with an unusual presentation of Kienböck's disease with symptoms similar to those of Vaughan-Jackson syndrome.

**Case presentation:**

A 40-year-old man of Indian ethnic origin with no known history of trauma presented to our clinic with a ten-day history of an inability to extend his right little and ring fingers with associated pain in his right wrist. He was being treated with long-term steroids but had no other significant medical history. His examination revealed an inability to extend the metacarpal and phalangeal joints of the right ring and little fingers with localized tenderness over the lunate bone. Spontaneous disruption of the extensor tendons was diagnosed clinically and, after radiological investigation, was confirmed to be secondary to dorsal extrusion of the fragmented lunate bone. The patient underwent surgical repair of the tendons and had a full recovery afterward.

**Conclusion:**

Kienböck's disease, though rare, is an important cause of spontaneous extensor tendon rupture. The original description of Vaughan-Jackson syndrome was of rupture of the extensor tendons of the little and ring fingers caused by attrition at an arthritic inferior radioulnar joint. We describe a case of a patient with Kienböck's disease that first appeared to be a Vaughan-Jackson-like syndrome.

## Introduction

Kienböck's disease is a condition of uncertain etiology that results in osteonecrosis of the carpal lunate bone. Patients with this disease present with reports of activity-related dorsal wrist pain, decreased wrist motion in the flexion-extension arc, and poor grip strength. The symptoms tend to occur more often in the dominant hand.

Vaughan-Jackson syndrome is characterized by the disruption of the digital extensor tendons, beginning on the ulnar side with the extensor digiti minimi (EDM) and extensor digitorum communis (EDC) tendon of the small finger. If the underlying pathology is not treated, sequential rupture of the ring, long, and index finger EDC tendons occurs [1]; ultimately, rupture of the extensor indicis proprius may follow [2]. Vaughan-Jackson's first report [1] of extensor tendon rupture described the cases of two elderly laborers with degenerative arthritis of the distal radioulnar joint.

Although rheumatoid arthritis is the most common underlying etiology of tendon rupture in the hand and wrist, abnormalities of the ulnar head resulting from either traumatic subluxation or Madelung's deformity, bony prominences, and local inflammatory changes resulting caused by both Kienböck's disease and calcium pyrophosphate dihydrate crystal deposition disease (pseudogout) have also been described as the cause of tendon rupture [3-7]. The ulnar side of the wrist is the most common site of extensor tendon rupture and is most often due to attritional changes caused by caput ulnar syndrome. We report an unusual case of a patient who came to our clinic with Kienböck's disease presenting as an attrition rupture of the extensor tendons to the ring and little fingers due to dorsal extrusion of the fragmented lunate.

## Case report

A 40-year-old man of Indian ethnic origin presented to our clinic without any history of trauma that he could recollect, an inability to extend the little and ring fingers of his right hand, and associated dorsal wrist pain of 10 days' duration. He had had Cushing's syndrome as a child and had undergone complete adrenalectomy in his teenage years. Since then, he had been on long-term steroid therapy and said he had no associated complications. He had no history of any other medical condition. He was right-hand-dominant and was unemployed at the time of presentation.

His physical examination revealed that he was unable to extend the metacarpal and phalangeal joints of his right ring and little fingers. Also, a small area of nodular elastic swelling associated with localized tenderness over the lunate bone was present over the dorsum of the proximal end of the space between the fourth and fifth metacarpal bones.

**Figure 1:**
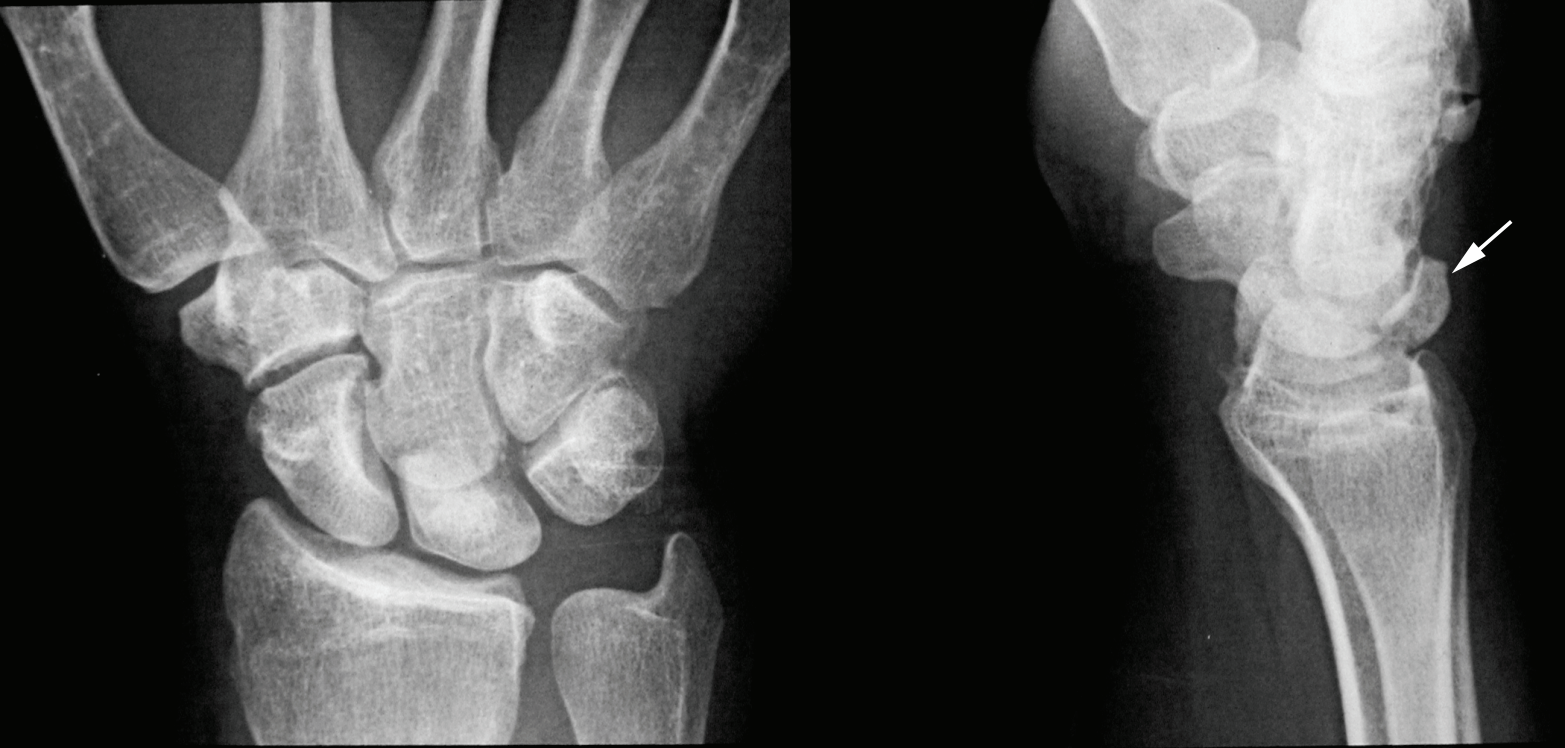
**Posteroanterior and lateral radiographs showing that the patient had Lichtman grade III Kienböck's disease with a large, displaced dorsal fragment**.

**Figure 2:**
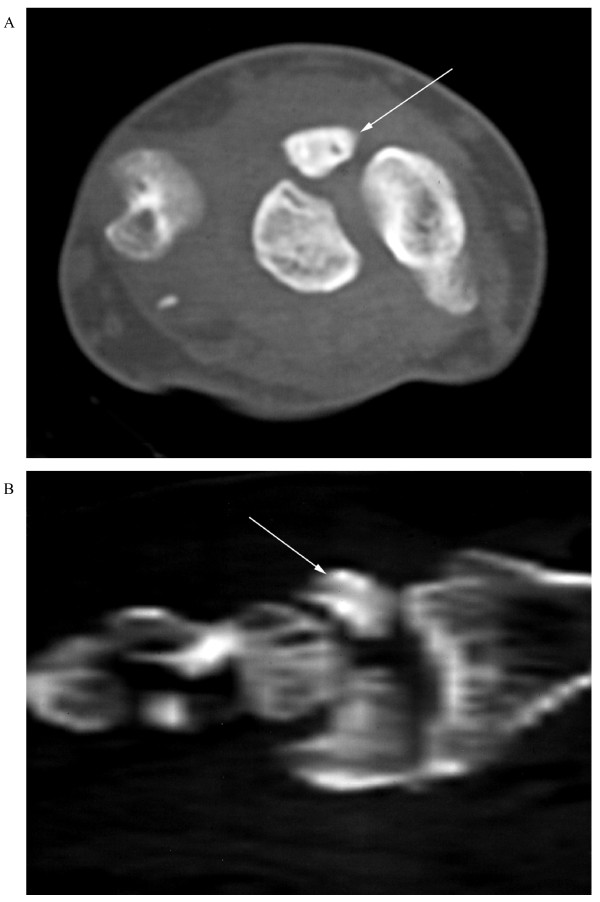
**Computed tomography scan showing reconstruction confirming the clinical diagnosis and radiological findings**.

A diagnosis of spontaneous rupture of the extensors to the ring and little fingers was made. Posteroanterior and lateral radiographs demonstrated Lichtman grade III Kienböck's disease with a large, displaced dorsal fragment (Figure 1). Computed tomography with reconstruction confirmed the clinical diagnosis and radiological findings (Figure 2).

He underwent surgical exploration through a straight dorsal incision. Rupture of the three extensor tendons was intimately related to a dorsal fragment of the lunate bone, which had become extruded through the dorsal capsule. The dorsal fragment was excised. The indicis proprius tendon was transferred to the little finger, and the ring finger tendon was cable-grafted to the EDC tendon of the middle finger. The patient's post-operative course was uneventful. He was subsequently treated with regular hand therapy and ultimately regained full functionality of his hand and wrist.

## Conclusions

Kienböck's disease, though rare, is an important cause of spontaneous extensor tendon rupture. Attrition rupture of tendons is a well-known problem. Finger tendon rupture has been related to various causes, but rarely has Kienböck's disease been implicated [8-11].

Murase *et al*. [12] and Ramkumar *et al*. [13] reported extensor tendon rupture and Pacha-Vicente *et al*. [14] reported attrition of EDM muscle following long-standing Kienböck's disease, but none of these authors reported these as the presenting complaints.

The original description of Vaughan-Jackson syndrome [1] was of the rupture of extensor tendons of the little and ring fingers caused by attrition at an arthritic inferior radioulnar joint. In this case report, we describe a patient with Kienböck's disease presenting as Vaughan-Jackson-like syndrome. Although the site of rupture was found to be directly related to the extruded fragment's causing mechanical attrition rupture, there may be a correlation between the use of long-term steroids and spontaneous rupture of the extensor tendons of the hand.

## Consent

Written informed consent was obtained from the patient for publication of this case report and any accompanying images. A copy of the written consent is available for review by the Editor-in-Chief of this journal.

## Competing interests

The authors declare that they have no competing interests.

## Authors' contributions

RR analyzed and interpreted the patient data. TM was a major contributor to the writing of the manuscript. Both authors read and approved the final manuscript.

## References

[B1] Vaughan-Jackson OJ Rupture of extensor tendons by attrition at the inferior radio-ulnar joint: report of two cases J Bone Joint Surg Br 1948 30B 528 530 18877990

[B2] Brooks P Extensor mechanism ruptures Orthopedics 2009 32 9 pii. 19751001 10.3928/01477447-20090728-31

[B3] Ducloyer P Leclercq C Lisfranc R Saffar P Spontaneous ruptures of the extensor tendons of the fingers in Madelung's deformity J Hand Surg (Br) 1991 16 329 333 10.1016/0266-7681(91)90064-u 1960504

[B4] Engkvist O Lundborg G Rupture of the extensor pollicis longus tendon after fracture of the lower end of the radius: a clinical and microangiographic study Hand 1979 11 76 86 10.1016/S0072-968X(79)80015-7 488782

[B5] Niwa T Uchiyama S Yamazaki H Kasashima T Tsuchikane A Kato H Closed tendon rupture as a result of Kienböck disease Scand J Plast Reconstr Surg Hand Surg 2010 44 59 63 20367065 10.3109/02844310903351301

[B6] Gladstone H Rupture of the extensor digitorum communis tendons following severely deforming fractures about the wrist J Bone Joint Surg Am 1952 24-A-3 698 700 14946225

[B7] Inoué G Attritional rupture of the extensor tendon due to longstanding Kienböck's disease Ann Chir Main Memb Super 1994 13 135 138 10.1016/S0753-9053(05)80386-2 7521659

[B8] James JI A case of rupture of flexor tendons secondary to Kienböck's disease J Bone Joint Surg Br 1949 31B 521 523 15408001

[B9] Lichtman DM Degnan GG Staging and its use in the determination of treatment modalities for Kienböck's disease Hand Clin 1993 9 409 416 8408251

[B10] Masada K Kawabata H Ono K Pathological rupture of flexor tendons due to longstanding Kienböck's disease J Hand Surg Am 1987 12 22 25 3805638 10.1016/s0363-5023(87)80154-5

[B11] Miki T Yamamuro T Kotoura Y Tsuji T Shimizu K Itakura H Rupture of the extensor tendons of the fingers: report of three unusual cases J Bone Joint Surg Am 1986 68 610 614 3957988

[B12] Murase T Ando Y Hiroshima K Extensor tendon rupture due to Kienböck's disease J Hand Surg Br 1997 22 597 598 10.1016/S0266-7681(97)80354-3 9752912

[B13] Ramkumar S Josty IC Sykes PJ Severe extensor tendon attrition and multiple tendon ruptures resulting from Kienböck's disease Ann Plast Surg 2000 45 647 650 10.1097/00000637-200045060-00014 11128766

[B14] Pacha-Vicente D Sevilla-Tirado J López-Martínez R Lluch-Bergadà A Mir-Bulló X Llusá-Pérez M Extensor digiti minimi damage due to longstanding Kienböck's disease J Hand Surg Eur Vol 2007 32 231 17222487 10.1016/J.JHSB.2006.11.012

